# Toward the Continuous Production of Multigram Quantities of Highly Uniform Supported Metallic Nanoparticles and Their Application for Synthesis of Superior Intermetallic Pt-Alloy ORR Electrocatalysts

**DOI:** 10.1021/acsaem.1c02570

**Published:** 2021-11-23

**Authors:** Luka Pavko, Matija Gatalo, Gregor Križan, Janez Križan, Konrad Ehelebe, Francisco Ruiz-Zepeda, Martin Šala, Goran Dražić, Moritz Geuß, Pascal Kaiser, Marjan Bele, Mitja Kostelec, Tina Đukić, Nigel Van de Velde, Ivan Jerman, Serhiy Cherevko, Nejc Hodnik, Boštjan Genorio, Miran Gaberšček

**Affiliations:** † Department of Materials Chemistry, National Institute of Chemistry, Hajdrihova 19, 1001 Ljubljana, Slovenia; ‡ Faculty of Chemistry and Chemical Technology, University of Ljubljana, 1001 Ljubljana, Slovenia; § ReCatalyst d.o.o., Hajdrihova 19, 1001 Ljubljana, Slovenia; ∥ Ami d.o.o., Trstenjakova 5, 2250 Ptuj, Slovenia; ⊥ Helmholtz-Institute Erlangen-Nürnberg for Renewable Energy (IEK-11), Forschungszentrum Jülich GmbH, Egerlandstr.3, 91058 Erlangen, Germany; # Department of Chemical and Biological Engineering, Friedrich-Alexander University Erlangen-Nürnberg, Egerlandstr. 3, 91058 Erlangen, Germany; % Department of Analytical Chemistry, National Institute of Chemistry, Hajdrihova 19, 1001 Ljubljana, Slovenia

**Keywords:** pulse combustion, continuous, double passivation, galvanic displacement, proton exchange membrane fuel cells (PEMFC), oxygen reduction reaction (ORR), gas-diffusion electrode (GDE)

## Abstract

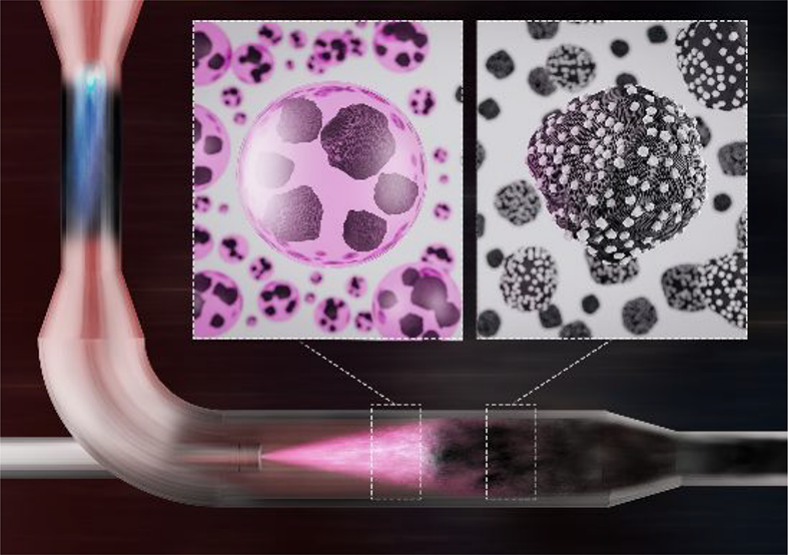

A fast and facile pulse combustion (PC) method that allows for the continuous production of multigram quantities of high-metal-loaded and highly uniform supported metallic nanoparticles (SMNPs) is presented. Namely, various metal on carbon (M/C) composites have been prepared by using only three feedstock components: water, metal–salt, and the supporting material. The present approach can be elegantly utilized also for numerous other applications in electrocatalysis, heterogeneous catalysis, and sensors. In this study, the PC-prepared M/C composites were used as metal precursors for the Pt NPs deposition using double passivation with the galvanic displacement method (DP method). Lastly, by using thin-film rotating disc electrode (TF-RDE) and gas-diffusion electrode (GDE) methodologies, we show that the synergistic effects of combining PC technology with the DP method enable production of superior intermetallic Pt–M electrocatalysts with an improved oxygen reduction reaction (ORR) performance when compared to a commercial Pt–Co electrocatalyst for proton exchange membrane fuel cells (PEMFCs) application.

## Introduction

Metallic nanoparticles (NPs) are spearheading the next-generation material science revolution, mainly in the fields of heterogeneous catalysis^1^ and electrocatalysis^2^ (e.g., proton exchange membrane fuel cells; PEMFCs) as well as in the fields of supercapacitors,^3^ sensors,^4^ and others.^5^ One can produce such a class of materials using various synthesis methods that can be categorized mainly into four different groups depending on the way they form the NPs: physical, chemical, a combination of both,^6^ or even bio-assisted methods.^7^

Following the NP synthesis step, for particular applications, there is often a need to immobilize these NPs on a supporting material (herein termed supported metallic NPs; SMNPs). Most commonly, the NP synthesis and deposition on the support material steps are subsequent in nature^7−10^ (two-step synthesis; usually also a third step of surfactant removal is required).^11^ However, in some cases, the deposition on the supporting material can also be achieved sequentially within the same synthesis step as the NP synthesis (one-step synthesis).^12−14^ While the desired properties of SMNPs vary from one application to another, the large variety of methods at hand allows the scientific community to tailor them in many ways according to the specific need. However, existing methods are often rather complex, poorly scalable, and time demanding and often require the use of expensive feedstock components as well as shape/size-controlling agents.

For instance, physical methods mainly operate on the top-down principle where high energy radiation, mechanical pressure, and electrical or thermal energy are applied to generate NPs out of the bulk materials.^15^ Relatively high energy input is necessary to break bonds and create the desired high surface area, which makes this approach inefficient. Furthermore, particle size distribution of the product is typically very broad, and it requires the use of surfactants to prevent NP agglomeration.

Chemical methods, on the other hand, typically utilize the bottom-up principle of synthesis, which allows for more precise NP tailoring. There exist several promising methods for industrial-scale production of SMNPs, such as the impregnation with chemical reduction or thermal decomposition.^13^ While this approach is rather straightforward, it does not allow for the most precise NP size and shape control. This is not the case for solvothermal (polyol) methods. The necessity of using high temperatures and pressures, long synthesis times, and complex organic solvents as well as reducing and size/shape-controlling agents makes such an approach rather expensive. Following the synthesis of NP, additional subsequent steps of NP deposition on the supporting material as well as surfactant removal are required, further complicating its industrial suitability for the production of SMNPs.^9,10^ Many of the above-mentioned approaches for synthesis of SMNPs thus fall in at least one of the mentioned pitfalls that were also recently highlighted by Kodama and co-workers in the latest *Nature Nanotechnology* review with respect to production of oxygen reduction reaction (ORR) Pt-based electrocatalysts for proton exchange membrane fuel cells (PEMFCs).^16^ Thus, when looking at both the practical and economical aspects for any given application, there is an inherent need for a more facile synthesis (e.g., water instead of an organic solvent) with a lower number of synthesis steps (e.g., simultaneous nature of NP synthesis and deposition, no requirements for surfactant removal). On top of that, the method should be inherently scalable and flexible to produce a high variety of products. As the “holy grail”, such a method would ideally also allow for a continuous production of SMNPs.

## Experimental Section

### Graphene Oxide Synthesis

Graphene oxide (GO) synthesis was based on a modified Hummer’s method described elsewhere.^17^ For this specific case, 1000 mL of sulfuric acid (96%, Carlo Erba) was added to a 5 L beaker. Slowly 110 mL of phosphoric acid (85%, Carlo Erba) was added. A PTFE anchor stirrer was centered to the middle and set to 150 rpm by using an IKA RW16 basic overhead stirrer. 30 g of graphite KS44 (Imerys) was slowly added to the mixture, followed by a slow addition of 1 wt equiv (∼30 g) of KMnO_4_ (Acros Organics) after 1 h of stirring. This has been repeated four more times every 24 h, adding up to total 5 wt equiv of KMnO_4_. Afterward, the mixture was left to stir for an additional 2 days. In the next step, under continuous stirring, the reaction mixture was quenched via direct addition of 3 L of ice, followed by slow addition of 30% H_2_O_2_ (Belinka) until the color changed from purple to yellowish. The stirring was then stopped, and GO was then left to settle on the bottom of the reaction mixture. This was followed by discarding of the supernatant that was replaced with fresh Milli-Q water (18.2 MΩ cm^–1^). Lastly, GO was subjected to several washing steps. In the first step, GO suspension was centrifuged for 30 min at 10500 rpm (Sorvall LYNX 4000, Thermo Scientific) to achieve sedimentation of GO and discard the supernatant. In the second step, GO was redispersed in 5% HCl prepared from 37% HCl stock solution (Carlo Erba) for 3 h to dissolve any residual metals. The mixture was centrifuged at 10500 rpm for 30 min to get rid of the supernatant. The last cleaning step composed of redispersing GO in Milli-Q water and soaking until the next day, followed by again centrifugation at 10500 rpm for 1 h to discard the supernatant. This has been repeated four more times, adding up to total of five washing cycles with Milli-Q. After the final supernatant was discarded, GO was again redispersed in Milli-Q water with an approximate concentration of ∼14 g_GO_ L^–1^_Milli-Q_ and treated with a homogenizer (Ultraturrax T-25 basic, IKA) for 1 h at maximum rpm setting.

### Pulse Combustion Reactor Synthesis

In the case of the synthesis of carbon black supported M/C composite materials, the feedstock suspension consisted of suspending 30 g of carbon black (Ketjen Black EC300J or Vulcan XC72) and a metal acetate hydrate in 1500 mL of Milli-Q water (18.2 MΩ cm^–1^). In the case of Co/C composite, 75.4 g of cobalt acetate tetrahydrate (Sigma-Aldrich) was used, in the case of Ni/C composite, 75.4 g of nickel acetate tetrahydrate (Sigma-Aldrich) was used, and in the case of Cu/C composite, 60.5 g of copper acetate monohydrate (Sigma-Aldrich) was used. For the synthesis of Cu/rGO composite, the feedstock suspension consisted of dissolving 21.0 g of copper acetate monohydrate (Sigma-Aldrich) in 1 L of ∼14 g_GO_ L^–1^_Milli-Q_ suspension. Lastly, all feedstock suspensions were mixed vigorously by using a homogenizer (Ultraturrax T-25 basic, IKA) for 10 min before being introduced to the PC reactor. Each feedstock suspension was then continuously stirred with a magnetic stirrer while being fed into the reactor by a peristaltic pump.

Specific details regarding the working principle of pulse combustion reactor have already been published elsewhere.^18−20^ In relation to this work, briefly, the materials were synthesized in a reactor setup consisting of a Helmholtz-type pulse combustor. Air is supplied to the combustor by a blower through an aerodynamic valve; the flow is measured by using a thermal mass flow meter. Fuel gas (propane) is supplied to the combustor at a constant flow and pressure by using a thermal mass flow meter and controller. According to the calculation of a stoichiometric quantity of air, a ratio between fuel and air can be set by varying the motor rotating speed of the blower or changing the fuel mass flow. Additionally, propane can be diluted with nitrogen to control the frequency and amplitude of pressure oscillations in the reactor. The frequency and amplitude influence the temperature and the composition of gases in the reactor. A higher amplitude means not only a lower temperature at the same frequency but also a more efficient burning of fuels. This in turn means that there is less oxygen present in the reacting hot zone. In the neck of the Helmholtz combustor there is a secondary gas inlet. This gas inlet is for maintaining or enhancing the reductive atmosphere by injecting acetylene into the stream of flue gases from the chamber of the combustor. The flow of acetylene is controlled with a thermal mass flow meter and controller. The neck of the combustor is coupled to the reactor pipe with a T-section that makes it possible to spray the precursors into the hot zone of the reactor, where thermal deposition of metals salts occurs and SMNPs are formed. Hot gas with SMNPs then travels through the cold zone, where temperature is maintained at ∼150 °C with indirect flow of cooling air. Lastly, the SNMPs reach the electrostatic filter where the product is collected.

### Synthesis of Dealloyed Intermetallic Pt-Alloy Electrocatalysts

#### Double Passivation with Galvanic Displacement

In the first step, Pt NPs were deposited onto the carbon support (Ketjen Black EC300J) via double passivation galvanic displacement method reported elsewhere.^12^ Briefly, the Pt deposition step consists of less noble metal (M) oxide passivation followed by carbon monoxide (CO) capping of Pt-based NPs formed by galvanic displacement of M after Pt-salt addition. Specifically, multigrams of M/C (M = Cu or Ni or Co; C = Ketjen Black EC300J) composites prepared by using the pulse combustion reactor were suspended in a slightly basic aqueous solution. The suspensions were then ultrasonicated (ultrasound bath Iskra Sonis 4) for 3 min (degassing). Afterward, the suspensions were first purged with Ar for 45 min and then switched to CO for 15 min while stirring with a magnetic stirrer. 0.1 M K_2_PtCl_4_ (Apollo Scientific) was added to the CO-saturated suspension of M/C composite with a syringe pump (WPI AL1000-220Z) while continuously purging the reaction mixture with CO. After the entire Pt–salt solution was added to the reaction mixture, the suspension was filtered and washed with Milli-Q water three more times. The obtained composites were left to dry at 50 °C overnight.

#### Formation of the Intermetallic Pt-Alloy

In the second step, Pt–Ni, Pt–Cu, and Pt–Co intermetallic alloys were formed via high-temperature thermal annealing of the obtained composites. All composite powders were placed in a Al_2_O_3_ crucible in a separate experiment due to different thermal annealing conditions. The crucibles were then put into a quartz tube that was sealed and purged with Ar for 2 h to ensure an inert atmosphere prior to raising the temperature. In the case of all experiments, the quartz tubes were purged with Ar for the entire duration of the thermal annealing process.

In the case of Pt–Ni/C electrocatalyst, the temperature was initially raised to 700 °C with a ramp of 10 K min^–1^ for 72 h. Afterward, the furnace was cooled to room temperature, followed by raising the temperature to 575 °C with a ramp of 10 K min^–1^ for another 72 h for the formation of the Pt–Ni intermetallic phase, followed by cooling to RT with a ramp of 3 K min^–1^.

In the case of Pt–Cu/C and Pt–Cu/rGO electrocatalysts, the temperature was initially raised to 800 °C with a ramp of 10 K min^–1^ for 1 h. Afterward, the furnace was cooled to room temperature, followed by raising the temperature to 575 °C with a ramp of 10 K min^–1^ for 3 h for the formation of the Pt–Cu intermetallic phase, followed by cooling to RT with a ramp of 3 K min^–1^.

Lastly, in the case of Pt–Co/C electrocatalyst, the temperature was initially raised to 700 °C with a ramp of 10 K min^–1^ for 24 h. Afterward, the furnace was cooled to room temperature, followed by raising the temperature to 600 °C with a ramp of 10 K min^–1^ for another 24 h for the formation of the Pt–Co intermetallic phase, followed by cooling to RT with a ramp of 3 K min^–1^.

#### Ex-Situ Chemical Activation (Dealloying)

All catalysts were subjected to the same activation (acid washing) protocol reported elsewhere.^21−23^ Briefly, the process involves a 24 h washing in 0.5 M H_2_SO_4_ at 80 °C. Afterward, the catalysts were washed four times with Milli-Q water (18.2 MΩ cm^–1^). The final electrocatalysts are denoted as d-int-Pt–Ni/C, d-int-Pt–Cu/C, d-int-Pt–Cu/rGO, and d-int-Pt–Co/C.

### ICP-OES and Digestion

All reagents used were of analytical grade or better. For sample dilution and preparation of standards, ultrapure water (18.2 MΩ cm^–1^, Milli-Q, Millipore) and ultrapure acids (HNO_3_ and HCl, Merck-Suprapur) were used. Standards were prepared in-house by dilution of certified, traceable, inductively coupled plasma (ICP)-grade single-element standards (Merck CertiPUR). A Varian 715-ES ICP optical emission spectrometer was used. Prior to ICP-OES analysis, each electrocatalyst was weighed (∼10 mg) and digested by using a microwave-assisted digestion system (Milestone, Ethos 1) in a solution of 6 mL of HCl and 2 mL of HNO_3_. Samples were then filtered, and the filter paper was again submitted to the same digestion protocol. These two times digested samples were cooled to RT and then diluted with 2% v/v HNO_3_ until the concentration was within the desired concentration range.

### X-ray Diffraction (XRD) Analysis

The powder XRD measurements of samples containing Ni and Cu were performed on a PANalytical X’Pert PRO MPD diffractometer with Cu Kα_1_ radiation (λ = 1.5406 Å) in the 2θ range from 10° to 60° with the 0.034° step per 100 s by using a fully opened X’Celerator detector. Samples were prepared on zero-background Si holder.

The powder XRD measurements of samples containing Co were performed on a PANalytical X’Pert PRO diffractometer with Cu Kα radiation (λ = 1.541874 Å) in the 2θ range from 10° to 60° with the 0.039° step per 300 s by using a fully opened Pixcel detector. Samples were prepared on a zero-background Si holder.

### Scanning Transmission Electron Microscopy (STEM) Analysis

STEM imaging was performed in a probe Cs-corrected scanning transmission electron microscope (Jeol ARM 200 CF) operated at 80 kV. Different regions of the samples were inspected to gather information from the most representative parts. For STEM analysis powder samples were transferred to lacey-carbon-coated copper or nickel grids.

### Raman Characterization

The Raman spectra were recorded in the spectral range from 50 to 3700 cm^–1^ by using an Alpha 300 RA confocal microscope (Witec, Ulm, Germany) with a 50× objective (0.8 NA). A green laser with an excitation wavelength of 532 nm was used with intensity ranging from ±0.1 to 1 mW. Integration times depended a bit on the stability of the signal and ranged from 5 to 20 s, depending on the sample. For each sample, three different locations were analyzed to verify the spectra.

### Electrochemical Evaluation via Thin Film Rotating Disc Electrode (TF-RDE)

#### Preparation of Thin Films and the Setup

Electrochemical measurements were conducted with a CompactStat (Ivium Technologies) in a two-compartment electrochemical cell in a 0.1 M HClO_4_ (Merck, Suprapur, 70%, diluted by Milli-Q, 18.2 MΩ cm^–1^) electrolyte with a conventional three-electrode system. Ag|AgCl was used as a reference and a graphite rod as a counter electrode. The working electrode was a glassy carbon disc embedded in Teflon (Pine Instruments) with a geometric surface area of 0.196 cm^2^. The Ag|AgCl reference was separated with a salt bridge to avoid Cl^–^ ions contamination. Prior to each experiment, the two-compartment electrochemical cell was boiled in Milli-Q water for 1 h, and the electrode was polished to mirror finish with Al_2_O_3_ paste (particle size 0.05 μm, Buehler) on a polishing cloth (Buehler). After polishing, the electrodes were rinsed and ultrasonicated (Ultrasound bath Iskra Sonis 4) in a Milli-Q/isopropanol mixture for 3 min. 20 μL of 1 mg mL^–1^ water-based well-dispersed electrocatalyst ink was pipetted on the glassy carbon electrode completely covering it and dried under ambient conditions. After the drop had dried, 5 μL of Nafion solution (ElectroChem, 5% aqueous solution) diluted in isopropanol (1:50) was added. The electrode was then mounted on the rotator (Pine Instruments).

For all electrocatalysts, the electrodes with electrocatalyst films were placed in the oxygen-saturated electrolyte without any potential control (at the OCP), and ORR polarization curves were measured at 1600 rpm in the potential window 0.05–1.0 V_RHE_ with a scan rate of 20 mV s^–1^ immediately after measurement of ohmic resistance of the electrolyte (determined and compensated for as previously reported).^24^ At the end of ORR polarization curve measurement, the electrolyte was purged with CO under the potentiostatic mode (0.05 V_RHE_) to ensure successful CO adsorption. Afterward, the electrolyte was saturated with Ar. CO electrooxidation was performed by using the same potential window and scan rate as in ORR, but without rotation and in an Ar-saturated electrolyte. After subtraction of background current due to capacitive currents, kinetic parameters were calculated at 0.95 V_RHE_ by using the Koutecky–Levich equation.^25^ The electrochemically active surface area (ECSA_CO_) was determined by integrating the charge in CO electrooxidation (“stripping”) experiments as described in ref (26). All potentials are given against the reversible hydrogen electrode (RHE), which was determined before the start of the experiment.

### Electrochemical Evaluation with Gas Diffusion Electrode (GDE)

#### Electrode Manufacturing

GDE manufacturing was done following the protocol used in previous work.^27^ The ink for the GDE fabrication composed of a total 1 wt % solids in a solvent mixture of 20 wt % isopropanol (IPA) in H_2_O. The solid fraction was composed of 30 wt % ionomer (Nafion D520; DuPont) and 70% d-int-Pt–M/C electrocatalyst, resulting in a gravimetric ionomer/carbon ratio of about 0.7. The ink was homogenized at 0 °C with an ultrasonic horn (Hielscher) at 60 W for 20 min. GDEs were fabricated by applying the catalyst ink onto a Freudenberg H23C8 gas diffusion media (230 μm thick) with an ultrasonic spraycoater (Biofluidix) on a heated stage at 85 °C. The ink flow rate and the movement speed of the spray head were controlled to a deposition rate of ∼6 μg_Pt_ cm^–2^ per deposition cycle. The Pt loading of the GDEs was measured by weighing (Sartorius Cubis, ±0.001 mg) the samples before and after the catalyst ink spray deposition.

#### Electrochemical Half-Cell and Instruments

An electrochemical half-cell was specially designed to conduct measurements on GDEs as described in detail in ref (27). For all electrochemical half-cell measurements, a VSP-300 (BioLogic) potentiostat mounted with two 2A booster boards was used.

#### Electrochemical Measurements Protocol

Before experiments with new catalysts or electrolyte, the half-cell was cleaned by boiling in 1% HNO_3_ solution (65% EMSURE, Merck) for 1 h. Afterward, it was boiled in ultrapure water (Milli-Q, Merck) five times. Before each experiment the cell was boiled two times in ultrapure water again. Between the experiments the cell was always stored in ultrapure water to avoid contamination. For the half-cell measurements 1 M HClO_4_ (70% Suprapur, Merck) was used as an electrolyte. The electrochemical active surface area (ECSA) was determined by integrating the CO stripping charge at a scan rate of 200 mV s^–1^. For ORR activity evaluation, galvanostatic steps were conducted both forward (−0.1 mA to −4.0 A) and backward (−4.0 A to −0.1 mA) consecutively. Thereby impedance was measured at each current step as previously reported.^27,28^ All experiments were conducted under ambient conditions (101 kPa, 20 °C) and repeated three times.

## Results and Discussion

In the present work, we present the pulse combustion (PC) technology as a method for continuous production of a wide variety of high-metal loaded, highly uniform SMNPs. In contrast to the decades of work on the flame-spray pyrolysis^29^ as current state-of-the-art continuous method for production of metallic nanoparticles, PC technology brings the most important breakthrough ingredient for production of SMNPs—the possibility of operating in a reductive atmosphere. Because flame-spray pyrolysis only allows one to operate in the oxidative environment, it is limited to the use of flammable precursors (such as, for example, alcoholates) and nonaqueous media as well only suitable for preparation of either unsupported metallic nanoparticles^30^ or thin films by using very high temperatures (1000–2000 K).^31^ On the other hand, by operating in slightly reductive conditions, PC technology combines all the positive aspects of both flame-spray pyrolysis^29^ and the pulsation-burning system,^32−34^ thus opening a completely new range of possibilities for production of SMNPs^18,20^ continuously at high scale, reproducibly, with a high metal loading, sub-5 nm average particle size as well as simple product flexibility for possible applications in electrocatalysis, heterogeneous catalysis, and sensors.

The first part of this work focuses to showcase the facile flexibility of PC technology on various carbon-supported metallic NPs composites (M/C; M = Cu, Ni or Co; C = commercial carbon blacks such as Ketjen Black EC300J, Vulcan XC72 as well as reduced graphene oxide = rGO). This has been achieved while using only three basic feedstock components: Milli-Q water as the solvent, metallic salt, and the carbon-based support forming the aqueous suspension. Consequently, carbon-based support, metallic loading, metallic NP size, and M type (or any combination thereof) were produced continuously at a significantly lower temperature with respect to flame-spray pyrolysis of only 450 °C and at a 20–45 g scale. In the second part of this work, we then provide one of the many use cases for the PC-prepared M/C composites in the synthesis of superior intermetallic Pt–M electrocatalysts for the application as ORR electrocatalysts in PEMFCs. More specifically, by preparation of metal precursors (M/C composites) using the PC technology, an important bottleneck related to galvanic displacement that has been limiting the past work^12,35−37^ has been solved. Thus, this study showcases the synergistic nature of the M/C composites prepared by using PC technology with the double passivation with the galvanic displacement method (DP method)^12,38^ for deposition of Pt NPs.^12,39^

**Figure 1:**
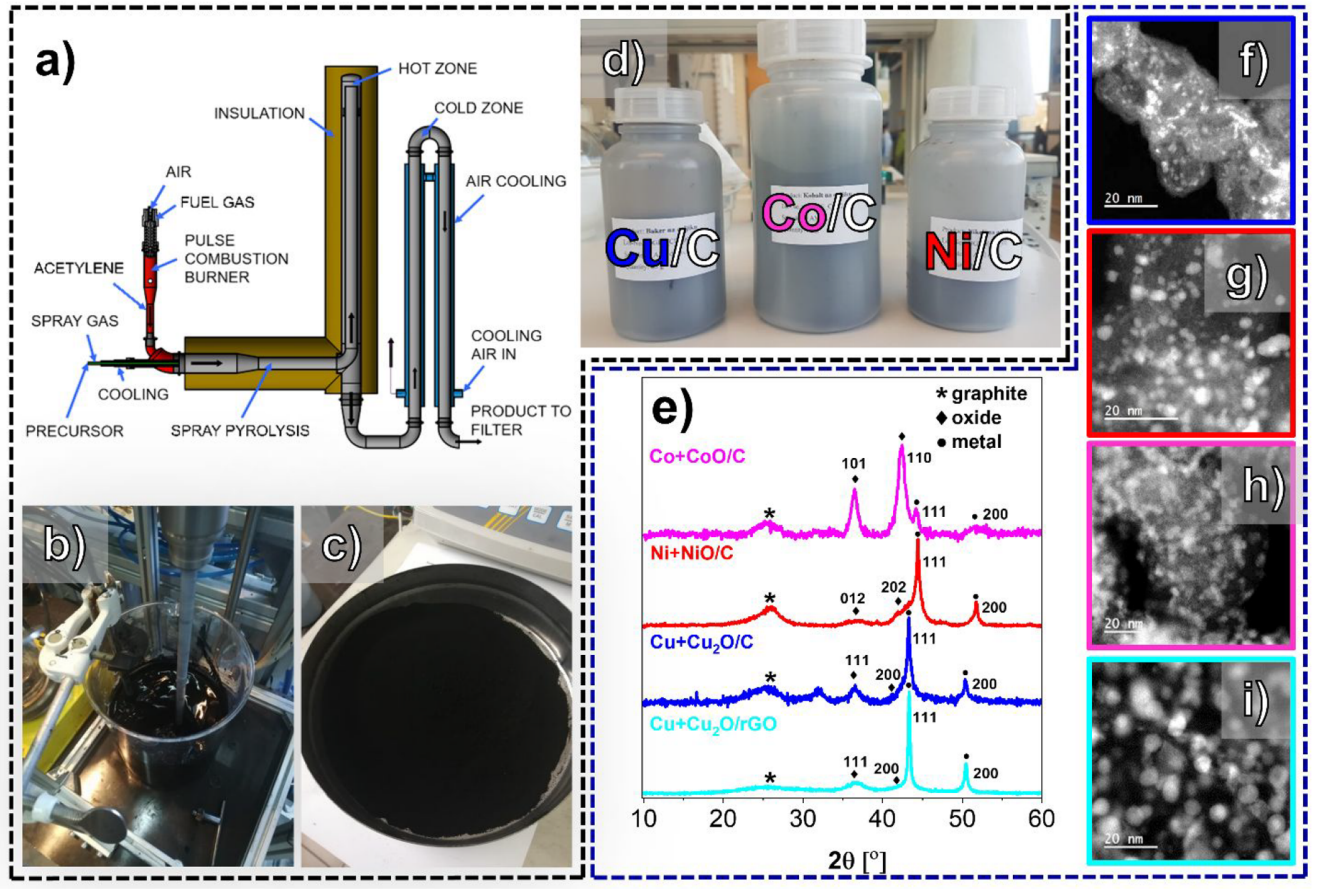
(a) Scheme of the current pilot-sized PC reactor, (b) image of an aqueous suspension of M-salt and carbon-based support, (c) image of a collected M/C composite product powder, and (d) image of the M/C (M = Cu, Ni, and Co) composites. (e) XRD spectra (the peak corresponding to the graphitic nature of the support as well as the most intense and visible peaks corresponding to the metallic and metal oxide phases are labeled with symbols) and (f–i) ADF STEM images of M/C composites, with the color code following that in (e). See also Figures S1–S6 for additional characterization (XRD, STEM, and Raman).

Despite the complexity of the proprietary reactor system, the production of the M/C composites is now, in fact, very facile. Figure 1a shows a scheme of the PC reactor used in this work. In the first step, the precursor suspension composed of only the metallic salt (specifically in the present work metal salt acetates), carbon-based support (carbon black or graphene oxide), and Milli-Q water (Figure 1b) is pumped into the reactor together with the spray carrier gas (nitrogen in the present work) by using a two-component nozzle that disperses the precursor suspension into ∼10 μm droplets. The small droplet size is important to achieve rapid evaporation of the solvent. The nozzle is parallel to the direction of the flue gas of the pulsation combustion burner that uses a mixture of air and fuel gas (propane in the present work). Furthermore, by addition of a very small amount of a reductive gas (acetylene in the present work) to the spray carrier gas at the spraying nozzle, any excess oxygen is consumed, enabling a slightly reductive atmosphere in the hot zone where the spray pyrolysis occurs. Thus, following the evaporation of the solvent in the hot zone, the metallic salt is thermally decomposed and/or reduced, resulting in formation of composites of carbon black or reduced graphene oxide (rGO) and sub-5 nm metallic NPs. In addition, slightly reductive conditions are of particular importance, preventing the M/C composite from burning and, thus, severe oxidation and sintering of the metallic NPs. The synthesized material is then cooled off from approximately 450 °C (hot zone) to 150 °C (cold zone) by using indirect air cooling (Figure 1a). Because the cooling is indirect, it is possible to reach a low enough temperature before the M/C composite powder gets in contact with air during product collection in the next step. This is again crucial to prevent the highly reactive metallic NPs from oxidizing or even resulting in burning of the carbon support. For the purpose of this study (a large variety of different products), we have used a “quasi-batch” approach by collecting (filtering) the M/C composite powders (Figure 1c) using an electrostatic filter. Regardless of using the “quasi-batch” approach, we have achieved product quantities of up to 45 g (Figure 1d). Important to note is also that this study is by no means showcasing the production limitations (in g h^–1^) of the presented PC methodology. For instance, by replacing the “quasi-batch” approach of product collection (using an electrostatic filter) with a bag filter, a production capacity increase of up to 300 g h^–1^ is achievable already on the current pilot-scale reactor (while also increasing the yields close to 100%). In addition, with just 10 times more powerful pulsation burner (100 kW instead of 10–11 kW in the current reactor), one could already produce on a much larger (3–20 kg h^–1^) scale.

Figure 1e (see also Figure S1) shows the X-ray diffraction (XRD) spectra of various M/C composites prepared by using the PC technology. In all the cases, we can observe a mixture of primarily metallic phases as well as a small amount of the metal oxide phase. Here it is important to note that initial optimization of air cooling was necessary to lower the temperature of the product in the transition from the hot to the cold zone (see Figure S1a). If the temperature at which the product came in contact with air was too high, the highly active high surface area metallic NPs would get oxidized or even initiate burning of the carbon-based support (which would result in NP sintering). However, this opens a possibility to tailor the degree of oxidation as well as the type of oxide (e.g., Cu_2_O/CuO or CoO/Co_3_O_4_). As already discussed, this can be achieved either via fine-tuning of the reductive gas mixture in the combustion chamber or with indirect air cooling in the transition from the hot to the cold zone. Furthermore, one is also not limited with a choice of the supporting material (see Figure S1b) nor with using only a single metal salt precursor (see Figure S1c).

Annular dark-field scanning transmission electron microscopy (ADF STEM) images (Figures 1f–i; see also Figures S2–S5) provide evidence on the NP dispersion and uniformity of the of the as-synthesized M/C composites using the PC reactor. We wish to remind the reader that these materials have been obtained at a very high multigram scale (up to 45 g per batch), reproducibly, as well as in a continuous production mode. This has been achieved without the need for any further optimization of the reactor parameters upon switching from one composite to another. Furthermore, in addition to the use of carbon black supports (see again the Supporting Information and Figure 1b), we have also prepared a composite on a reduced graphene oxide support (rGO). In the case of Cu/rGO composite, the only difference was that instead of the carbon black, a graphene oxide (GO) material prepared via the modified Hummers method^17^ from a graphite precursor was used. Thus, in the case of Cu/rGO composite, in addition to the thermal reduction of the metal salt and deposition of metallic NPs, thermal reduction of GO into rGO occurs simultaneously as well. To be sure that the used carbon-based supports have not been modified during the synthesis of M/C composites in the PC reactor, Raman spectroscopy was used to compare the pristine carbon-based material with the M/C composite (see Figure S5). We observe that the *I*_D_/*I*_G_ remains unchanged even after the carbon-based support is processed in the PC reactor. Interestingly, both GO and the Cu/rGO composite exhibit a lower *I*_D_/*I*_G_ (below 1 for rGO and above 1 for Ketjen Black EC300J) than all the M/C composites on Ketjen Black EC300J. This suggests that perhaps both GO and rGO have less structural defects in contrast to the partly graphitized Ketjen Black EC300J.^40^

**Figure 2:**
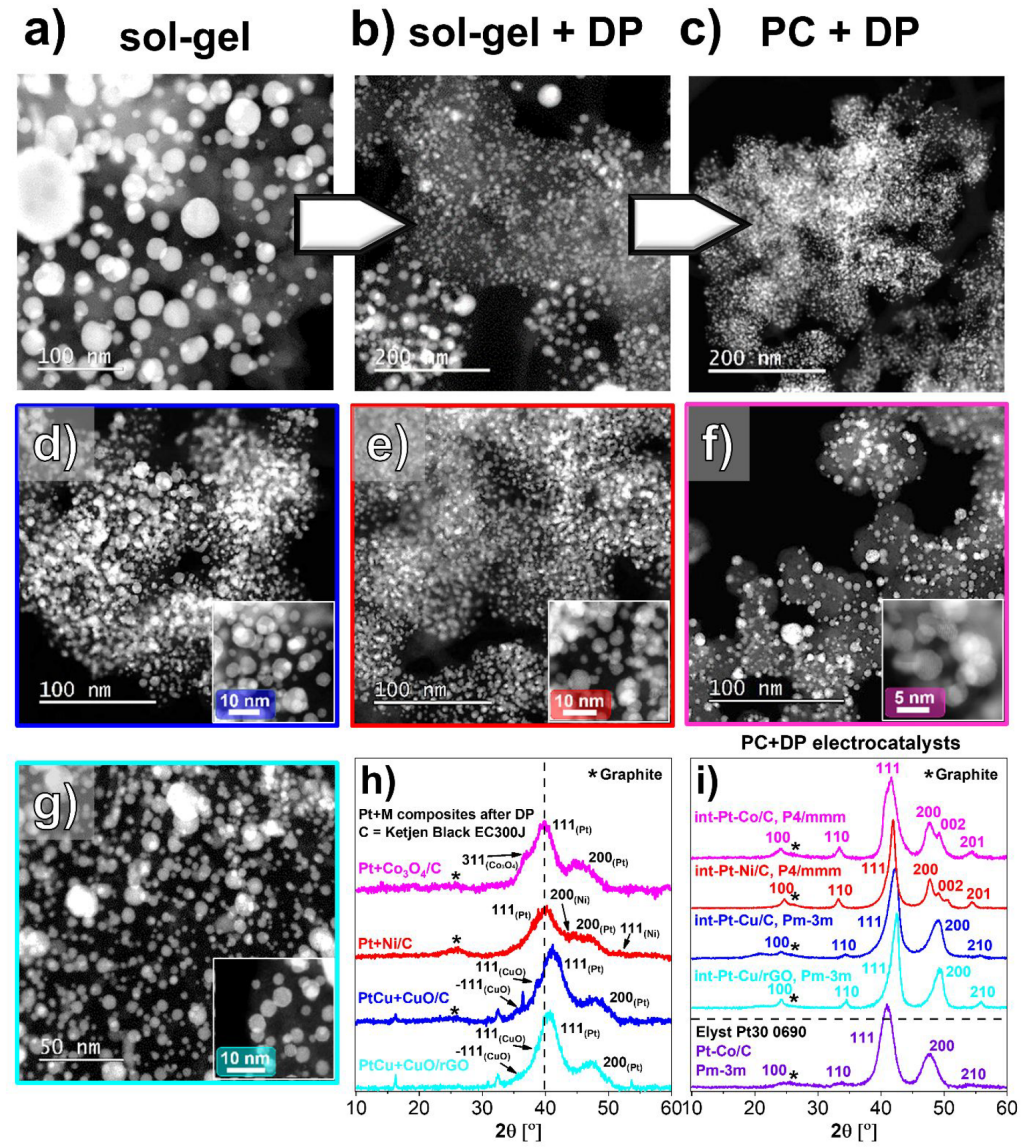
Improvement in the dispersion of Pt–M NPs over carbon upon transitioning from the (a) sol–gel method for preparation of M/C composites with conventional galvanic displacement for deposition of Pt NPs,^43−46^ (b) combining sol–gel for preparation of M/C composites with DP method for Pt NP deposition,^12,39^ and (c) utilizing the synergy between PC methodology for the production of M/C composites in combination with DP method for Pt NP deposition (this work).^12,39^ (d–g) ADF STEM images of the final d-int-Pt–Cu/C, d-int-Pt–Ni/C, d-int-Pt–Co/C, and d-int-Pt–Cu/rGO electrocatalysts. XRD spectra of (h) Pt + M/C composites (see also Figures S7–S10 for additional STEM characterization), and (i) thermally annealed int-Pt–M/C electrocatalysts (see also Figures S11–S14 for additional STEM characterization). The thermally annealed PC + DP electrocatalysts are compared to a commercial Pt–Co/C reference from Umicore (Elyst Pt30 0690).

In the second part of this work, we present one of the many use cases for the M/C composites prepared by using the PC technology by transforming them into superior intermetallic Pt-alloy electrocatalysts for the sluggish ORR in PEMFCs.^41,42^ Briefly, following the production of M/C composites (Figure 1), the intermetallic Pt-alloy PEMFC electrocatalysts presented in this study (Figure 2) were synthesized in a three-step process. In the first step, Pt-based NPs were deposited on the M/C composites by using our proprietary DP method.^12,38^ The method allows for facile flexibility in the final electrocatalyst design in terms of metal loading (Pt + M), chemical composition (Pt:M), choice of carbon support, and choice of M (see Figures S7–S10 for STEM characterization of Pt + M composites on Ketjen Black EC300J and rGO).^12,39^ In addition, the presence of the less noble metal oxides (Figure 1e) is in fact one of the criteria for the DP method mechanism to occur as it blocks direct deposition of Pt on the sacrificial metal M, which is not the case when using the conventional galvanic displacement.^43−46^ Thus, this has been the main reason for not attempting to further optimize the reductive atmosphere conditions in the combustion chamber of the PC reactor to achieve complete reduction of the metallic NPs in this study. In the second step, the obtained Pt-containing composites were subjected to a high-temperature thermal annealing to form an intermetallic Pt-alloy structure.^35^ For the last step, all Pt–M electrocatalyts were subjected to a dealloying step^22,47^ and, thus, the formation of a Pt-rich overlayer. In continuation, these final thermally annealed and activated electrocatalysts will be denoted as d-int-Pt–M/C (M = Cu, Ni, or Co) or d-int-PtCu/rGO.

For further context to the reader, we have based our past approach on preparing M/C composites using a modified sol–gel method.^48,49^ While multigram quantities were achievable, there were many limitations: (i) preparation of multigram quantities took weeks rather than minutes as in the case of PC technology, (ii) the reaction mixtures were much more complex while PC boils it down to only the basic three components in the feedstock (Milli-Q water, metal salt, and carbon support), (iii) M particles were highly polydisperse (20–200 nm with also micrometer-sized particles in contrast to few nanometers in the case of M/C composites prepared with the PC reactor), and (iv) compatibility with different M (as well as compatibility with GO) was poor. The PC methodology alleviated all of the above-mentioned limitations by enabling a subsequent increase in M loading, while decreasing and narrowing the average particle size down to merely a few nanometers. This is important because in the case of galvanic displacement the sacrificial metal acts as the “reducing agent” for the Pt-salt. Thus, having more M as well as a higher surface area of M is equal to having a higher amount of “reducing agent”.

Figures 2a–c showcase the improvement in the dispersion of Pt–M NPs of already thermally annealed^35^ Pt–M electrocatalysts upon transitioning from the use of the sol–gel method produced M/C composites in combination with regular galvanic displacement for Pt NPs deposition toward the present PC + DP approach. Figure 2a shows Pt deposition on sol–gel precursor using the regular galvanic displacement method (large Pt–M particles).^48,49^ On the other hand, if the DP method is used instead of regular galvanic displacement (sol–gel + DP), one already notices a significant improvement in dispersion of Pt–M NPs as shown in Figure 2b, with much smaller Pt–M NPs but also still some less homogeneous areas.^12^ Finally, as presented in this work, by combining the synergy of both the DP method and PC produced M/C composites (PC + DP), incredibly high uniformity and density of the Pt–M NPs are achieved as shown in Figure 2c. This shows a general trend that a better dispersion of as-deposited Pt NPs also results in a more homogeneous dispersion of alloy Pt–M NPs upon exposure to the high temperature conditions required to obtain the Pt–M intermetallic crystal structure (see also Figure S15 for a particle size distribution comparison).

The flexibility of our Pt–M electrocatalyst preparation is presented in Figures 2d–g, which show ADF STEM images at two magnifications of the final thermally annealed and dealloyed d-int-Pt–Cu/C, d-int-Pt–Ni/C, d-int-Pt–Co/C, and d-int-Pt–Cu/rGO electrocatalysts (see also Figures S11–S14 for additional STEM characterization). In all cases, the density of Pt–M NPs and the uniformity are notably very high. For the sake of a more complete description, XRD spectra of the Pt + M/C composites obtained after the Pt-based NP deposition step following the DP method using M/C composites (Figure 2h), and the corresponding thermally annealed Pt-alloy electrocatalysts (Figure 2i) are also provided. Prior to thermal annealing (Figure 2h), PC + DP electrocatalysts exhibit very broad peaks corresponding to a very small Pt NP size with a cubic (*Fm*3̅*m*) crystal structure including some leftover M oxide. Upon alloying (Figure 2i), all PC + DP electrocatalysts show the presence of the intermetallic structure (tetragonal *P*4/*mmm* phase in the case of Pt–Co and Pt–Ni and cubic *Pm*-3*m* in the case of Pt–Cu). This subclass of alloys—intermetallics—has already caught much attention in the past due to its promising stability and activity benefits.^14,48,50−53^ Because of their different crystal symmetry, intermetallic alloys, when compared to their disordered counterparts, show extra peaks in XRD, which are usually termed superlattice ordering peaks.^54^ In both cases (Figures 2h,i), the XRD spectra are in a good agreement with what has been observed on STEM (Figures 2d–f; see also Figures S7–S14).

**Figure 3:**
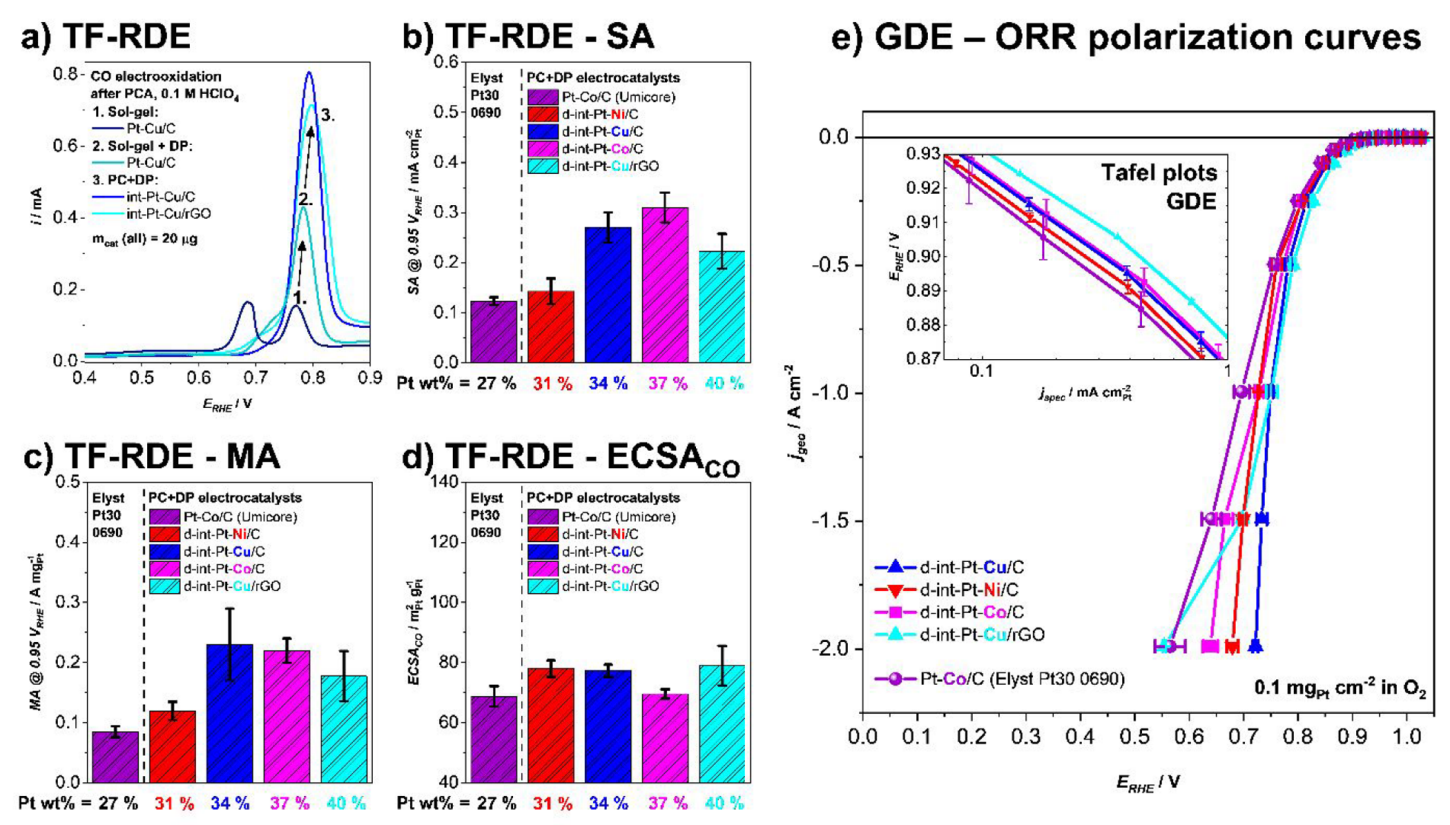
(a) Comparison of CO electrooxidation CVs (0.1 M HClO_4_, no rotation, Ar saturated after CO adsorption, 20 mV s^–1^) of Pt–Cu electrocatalysts obtained with (1) sol–gel or a combination of (2) sol–gel + DP or (3) PC + DP. TF-RDE comparison of final activated PC + DP electrocatalysts with the commercial Pt–Co/C electrocatalyst from Umicore (Elyst Pt30 0690): (b) SA (at 0.95 V_RHE_), (c) MA (at 0.95 V_RHE_), and (d) ECSA_CO_. See also Figures S16–S18 for CO electrooxidation CVs and ORR polarization curves measured in liquid electrolyte TF-RDE. (e) High current density performance of the final activated PC + DP electrocatalysts with the commercial Pt–Co/C electrocatalyst from Umicore (Elyst Pt30 0690) measured in solid electrolyte GDE.

To prove the suitability and applicability of the PC+DP synthesized composites for electrochemical application as ORR electrocatalysts, we initially compared their performance against the commercial Pt–Co/C electrocatalyst from Umicore (Elyst Pt30 0690) by using the thin-film rotating disc electrode methodology (TF-RDE;^36,37^Figures 3a–d). TF-RDE is a widespread conventional half-cell method used for preliminary studies of kinetic ORR performance at low current densities (preliminary activity screening; specific activity = SA and mass activity = MA) as well as evaluation of the electrochemically active surface area (ECSA). The Pt surface area can be determined via the charge during CO-electrooxidation (ECSA_CO_).^55^Figure 3a shows a comparison of CO-electrooxidation CVs measured with TF-RDE of Pt–Cu alloy electrocatalysts prepared with a combination of (1) sol–gel with a conventional GD (Figure 2a), (2) sol–gel + DP (Figure 2b), and (3) PC + DP (Figure 2c). One can see that despite the use of the same catalyst loading (∼100 μg_cat_ cm^–2^) for the measurements of all electrocatalysts, the area of the peak at ∼0.8 V_RHE_ corresponding to CO-electrooxidation increases significantly in the order of sol–gel (Figure 2a) ≪ sol–gel + DP (Figure 2b) ≪ PC + DP (Figure 2c). Furthermore, the similarity between peak areas of int-Pt–Cu/C and int-Pt–Cu/rGO indicates that a comparable quality of Pt–Cu alloy NP dispersion can also be achieved by using a completely different supporting material and thus adding to the flexibility of our approach. In addition, since the peak area of CO-electrooxidation directly correlates to ECSA_CO_, this comparison reveals that besides the visual improvement in homogeneity observed on STEM (Figures 2b,c), the synergy of PC + DP also translates to a much higher electrochemical Pt surface area (Figure 3a). Figures 3b–d (see also Figures S16–S18 for CO-electrooxidation CVs and ORR polarization curves) compare the kinetic performance (SA and MA) as well as the Pt surface area (ECSA_CO_) of the final dealloyed PC + GD electrocatalysts in comparison to the commercial Pt–Co/C benchmark from Umicore (Elyst Pt30 0690). We can see that all our electrocatalysts exceed that of the Pt–Co reference in both the SA and MA as well as ECSA_CO_. Here, it is worth also considering that a higher Pt surface area (ECSA_CO_) is achieved despite PC + DP electrocatalysts having a higher loading of Pt over carbon (in wt %). This is important because with an increasing metal loading, a decrease in Pt utilization is expected due to a higher difficulty of achieving quality dispersion of the metal and, thus, a higher degree of agglomeration.^56^

After this preliminary activity screening with TF-RDE, the final PC + DP electrocatalysts were also evaluated at the high mass-transport region by using a GDE half-cell approach.^57,58^ This method has been proposed as a suitable tool to bridge the gap between the fundamental electrochemical catalyst evaluation and applied fuel cell research in single cells.^28,57,59,60^ A very low Pt loading of only 0.1 mg_Pt_ cm^–2^ was used for all measurements, which is in accordance with DoE 2025 targets.^61^ Once again, the electrocatalysts were compared to the same commercial Pt–Co/C electrocatalyst from Umicore (Elyst Pt30 0690). The GDE half-cell measurements reveal that also in relevant current and potential ranges for fuel cell applications all final PC + DP electrocatalysts show a higher performance compared to the commercial benchmark (Figure 3e). With potentials >0.7 V_RHE_ up to current densities of 2 A cm^–2^, the d-int-Pt–Cu/C electrocatalyst has the highest activity of all tested catalysts in the mass-transport region. The d-int-Pt–Cu/rGO electrocatalyst exhibits the highest activity in the kinetic region (see GDE Tafel plot in Figure 3e). However, at current densities >1 A cm^–2^ mass transport limitations start to affect the rGO supported electrocatalyst performance. This can be attributed to either the less porous carbon support or a nonideal catalyst layer structure due to nonoptimized ink formulation.^62^ For the sake of comparability, the same ink composition was used for all the different catalysts. An intensive examination of the impact of ink formulation and key rGO properties on the catalyst performance will be the subject of upcoming work.

## Conclusion

In conclusion, we presented the pulse combustion technology that opens the possibility for continuous high-scale production of a wide variety of SMNPs under slightly reductive conditions that are otherwise susceptible to irreversible oxidation. As part of this work, we have focused mainly on M/C composites (M = Cu, Co, or Ni; C = commercial carbon blacks or rGO). In addition, we have shown the possibility of varying the carbon-based support, metallic loading, metallic NP size, and M type (or any combination thereof). In the second part we presented a use case for M/C composites by preparing Pt-alloy oxygen reduction reaction electrocatalysts for the use in proton exchange membrane fuel cells. For that we have combined the synergistic effects of the pulse combustion synthesized M/C composites as metal precursors and the double passivation with the galvanic displacement method used for deposition of Pt NPs. The synergy between both methods resulted in a very uniform dispersion of intermetallic Pt-alloy NPs. Lastly, the final PC + DP electrocatalysts were evaluated by using two different electrochemical half-cell methods. Specifically, a liquid electrolyte thin-film rotating disc electrode was used for evaluation of the kinetic performance as well as the electrochemically active surface area, while the solid electrolyte gas-diffusion electrode was used for evaluation of the performance at higher current densities. In comparison to the commercial Pt–Co/C electrocatalyst from Umicore (Elyst Pt30 0690), the final PC + DP electrocatalysts exhibited an increased performance measured in TF-RDE in terms of both the specific activity and mass activity as well as in the electrochemically active surface area. Most importantly, GDE characterization showed that compared to the state-of-the-art commercial benchmark, all final PC + DP electrocatalysts in addition also exhibited a higher performance at the industrially relevant high current densities. This provides a high promise in terms of industrial applicability of the pulse combustion method. Lastly, such an approach can be easily applied for the synthesis of the supported metal or metal oxide NPs for numerous other applications in electrocatalysis as well as applications involving heterogeneous catalysis and sensors.

## Supporting Information Available

Additional XRD spectra comparison, ADF and BF STEM images, Raman spectra comparison of carbon-based materials, particle size distribution of nanoparticles, electrochemical measurements which include CO-electrooxidation CVs and ORR polarization curves (PDF)
