# Supplementary material for: Toward the Continuous Production of Multigram Quantities of Highly Uniform Supported Metallic Nanoparticles and Their Application for Synthesis of Superior Intermetallic Pt-Alloy ORR Electrocatalysts
Source: ACS Appl Energy Mater. 2021 Nov 23;4(12):13819–29. doi: 10.1021/acsaem.1c02570 (PMC8715446; doi:10.1021/acsaem.1c02570)
Supplement: Supplementary file 1 — ae1c02570_si_001.pdf [file ae1c02570_si_001.pdf]

Supporting information

Toward the Continuous Production of Multigram Quantities of Highly Uniform Supported Metallic Nanoparticles and Their Application for Synthesis of Superior Intermetallic Pt-Alloy ORR Electrocatalysts

Luka Pavko[a,b,‡], Matija Gatalo[a,c,‡,], Gregor Križan[d], Janez Križan[d], Konrad Ehelebe[e,f], Francisco Ruiz-Zepeda[a], Martin Šala[g], Goran Dražić[a], Moritz Geuß[e,f], Pascal Kaiser[e,f], Marjan Bele[a], Mitja Kostelec[a,b], Tina Đukić[a,b], Nigel Van de Velde[a], Ivan Jerman[a], Serhiy Cherevko[e], Nejc Hodnik[a], Boštjan Genorio[b], Miran Gaberšček[a,*]*

[a] Department of Materials Chemistry, National Institute of Chemistry, Hajdrihova 19, 1001 Ljubljana, Slovenia

[b] Faculty of Chemistry and Chemical Technology, University of Ljubljana, 1001 Ljubljana, Slovenia

[c] ReCatalyst d.o.o., Hajdrihova 19, 1001 Ljubljana, Slovenia

[d] Ami d.o.o., Trstenjakova 5, 2250 Ptuj, Slovenia

[e] Helmholtz-Institute Erlangen-Nürnberg for Renewable Energy (IEK-11), Forschungszentrum Jülich GmbH, Egerlandstr.3, 91058 Erlangen, Germany

[f] Department of Chemical and Biological Engineering, Friedrich-Alexander University Erlangen-Nürnberg, Egerlandstr. 3, 91058 Erlangen, Germany

[g] Department of Analytical Chemistry, National Institute of Chemistry, Hajdrihova 19, 1001 Ljubljana, Slovenia

[‡] these authors contributed equally to this work

* to whom correspondence should be addressed: matija.gatalo@ki.si, miran.gaberscek@ki.si

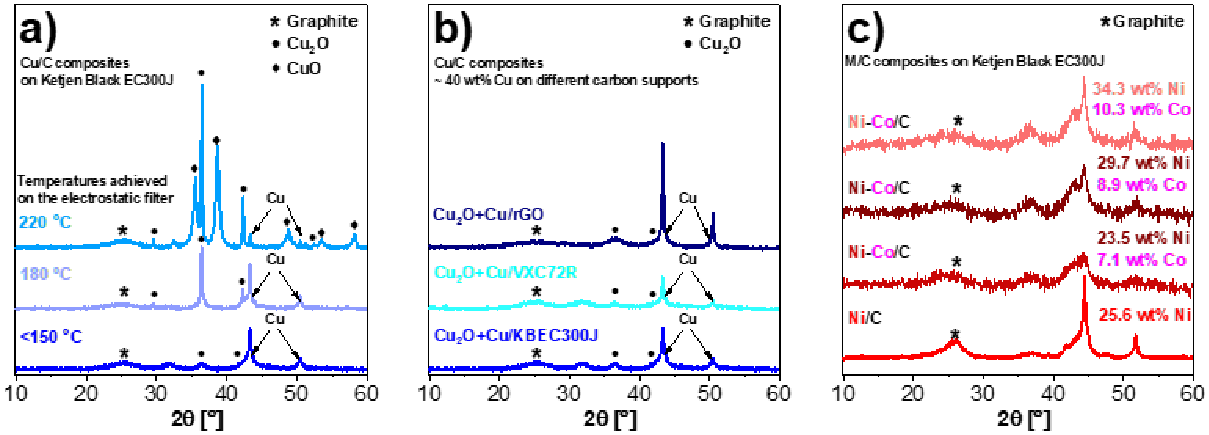

Figure S1. (a) XRD spectra comparison of Cu/C composites obtained at various temperatures at the electrostatic filter of the pulse combustion reactor. **(b)** XRD spectra comparison of Cu/C composites on various carbon-based supports prepared *via* the pulse combustion method. **(c)** XRD spectra comparison of Ni-Co composites (mix of two metals) with various metal loadings over carbon prepared *via* the pulse combustion method.[1]

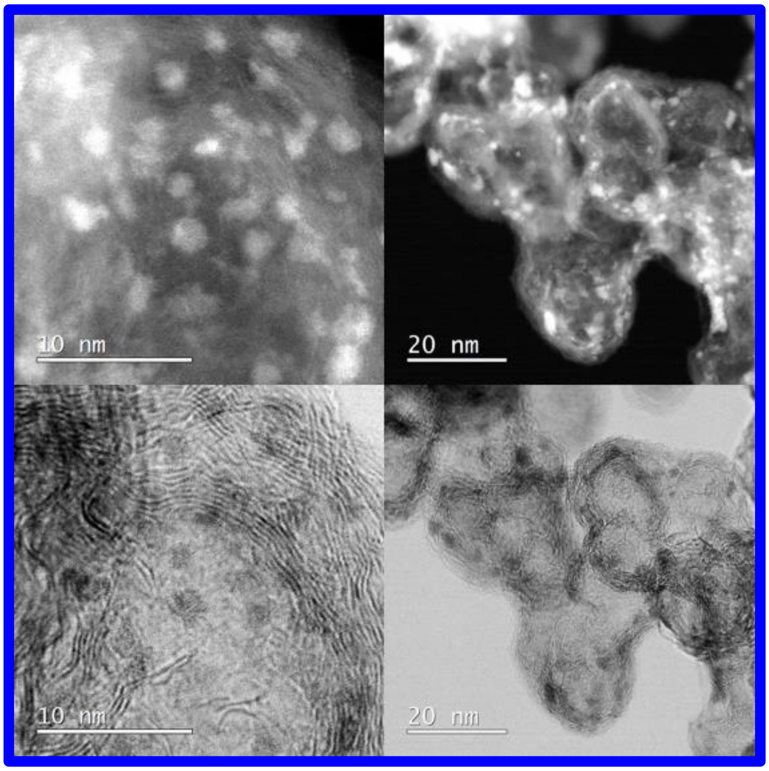

Figure S2. ADF and BF STEM images of Cu+Cu_2O/C (C = Ketjen Black EC300J) composite prepared *via* the pulse combustion method.[1]

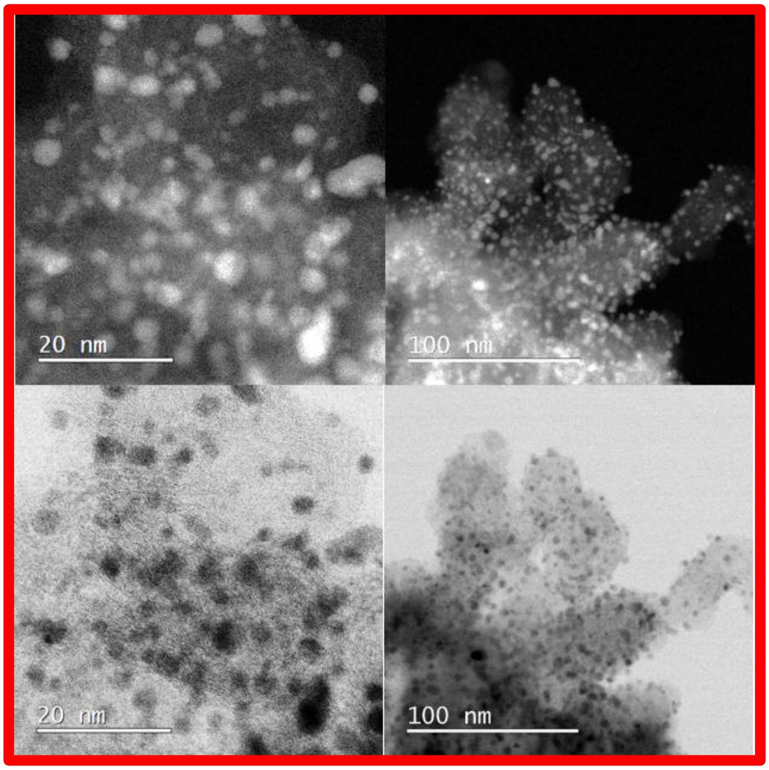

Figure S3. ADF and BF STEM images of Ni+NiO/C (C = Ketjen Black EC300J) composite prepared *via* the pulse combustion method.[1]

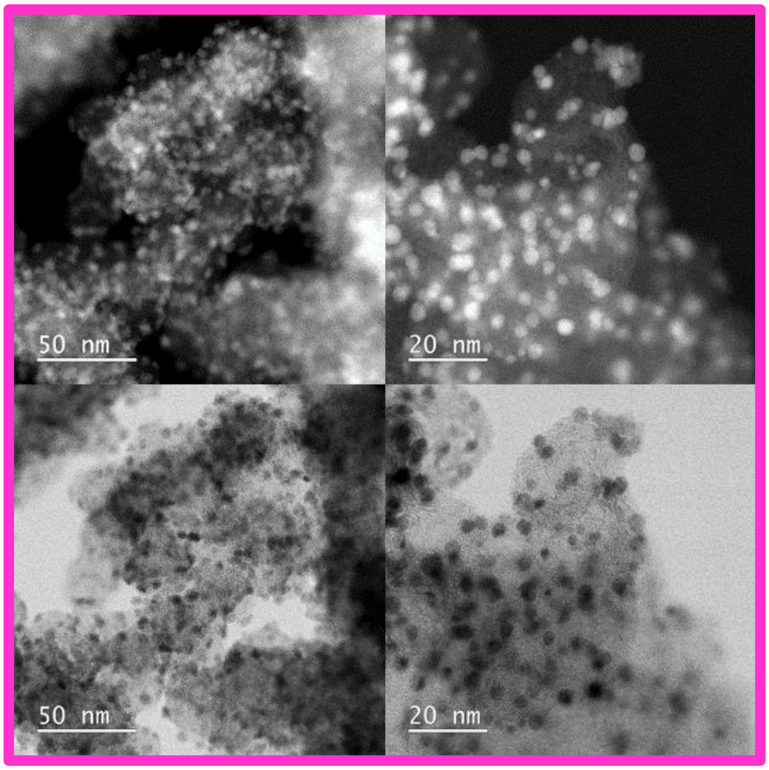

Figure S4. ADF and BF STEM images of Co+CoO/C (C = Ketjen Black EC300J) composite prepared *via* the pulse combustion method.[1]

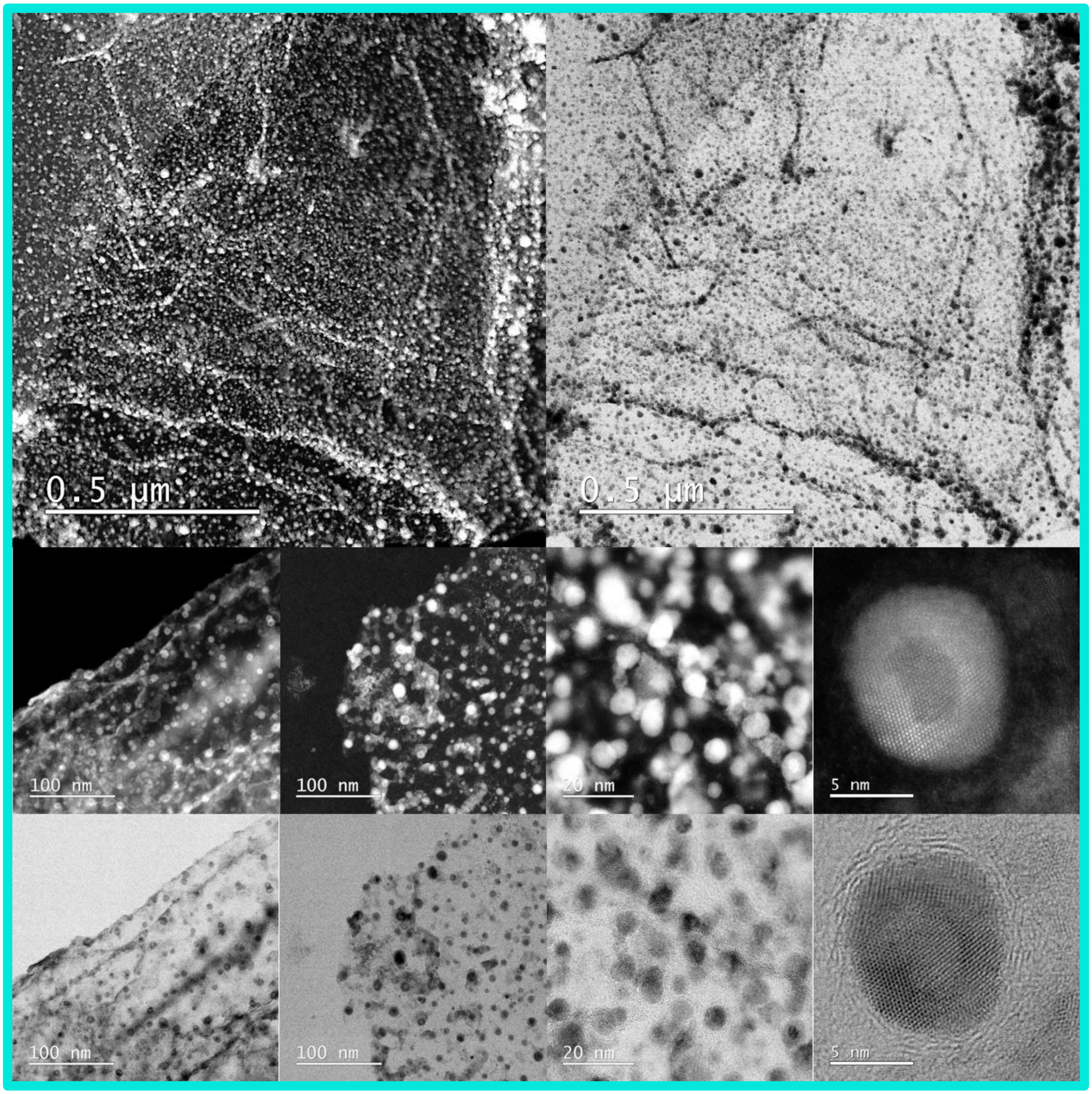

Figure S5. ADF and BF STEM images of Cu+Cu_2O/rGO composite prepared *via* the pulse combustion method.[1]

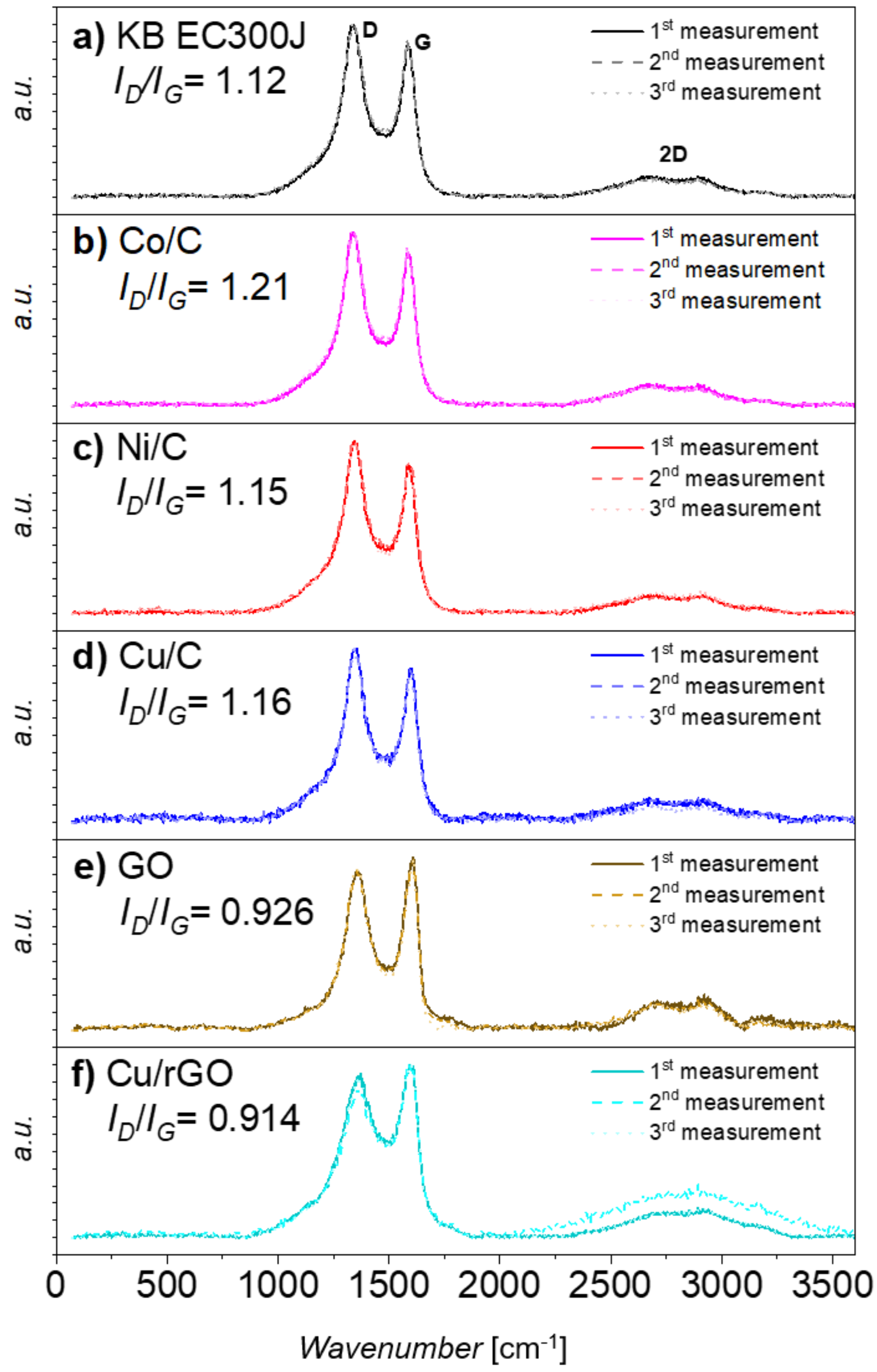

Figure S6. (a-f) Comparison of Raman spectra of starting carbon-based supports (Ketjen Black EC300J and GO) and corresponding M/C (M = Co, Ni and Cu) & M/rGO composites prepared *via* the pulse combustion method at 3 different locations for reproducibility.

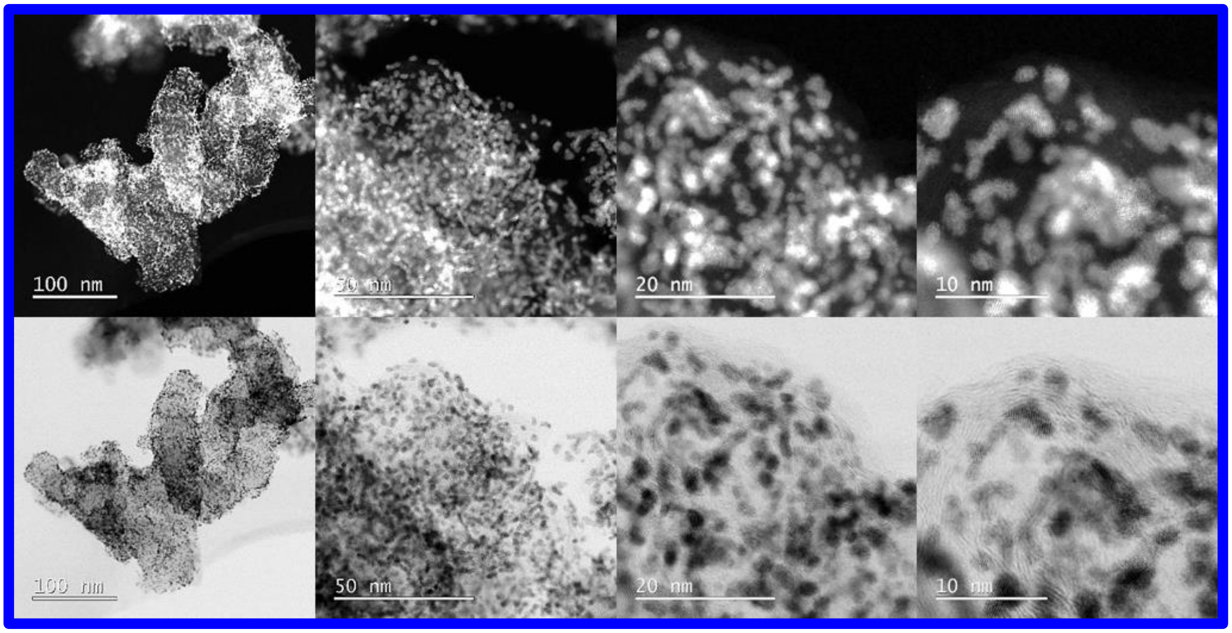

Figure S7. ADF and BF STEM images of PtCu+CuO/C (C = Ketjen Black EC300J) after Pt NP deposition step *via* GD method.[1]

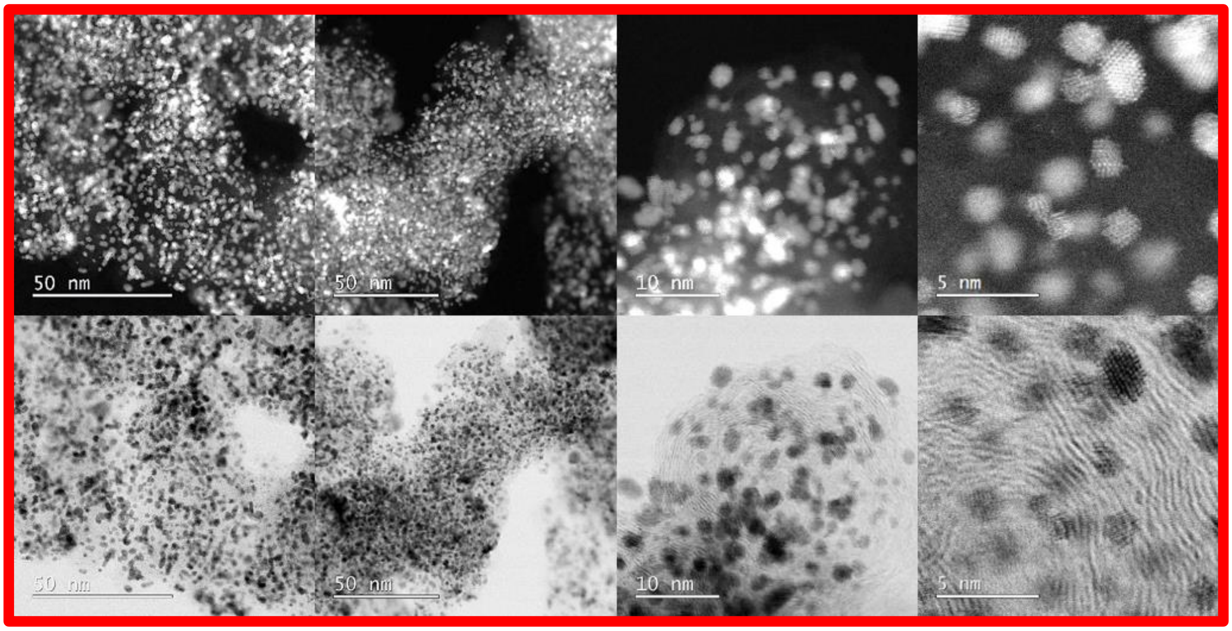

Figure S8. ADF and BF STEM images of Pt+Ni/C (C = Ketjen Black EC300J) after Pt NP deposition step *via* GD method.[1]

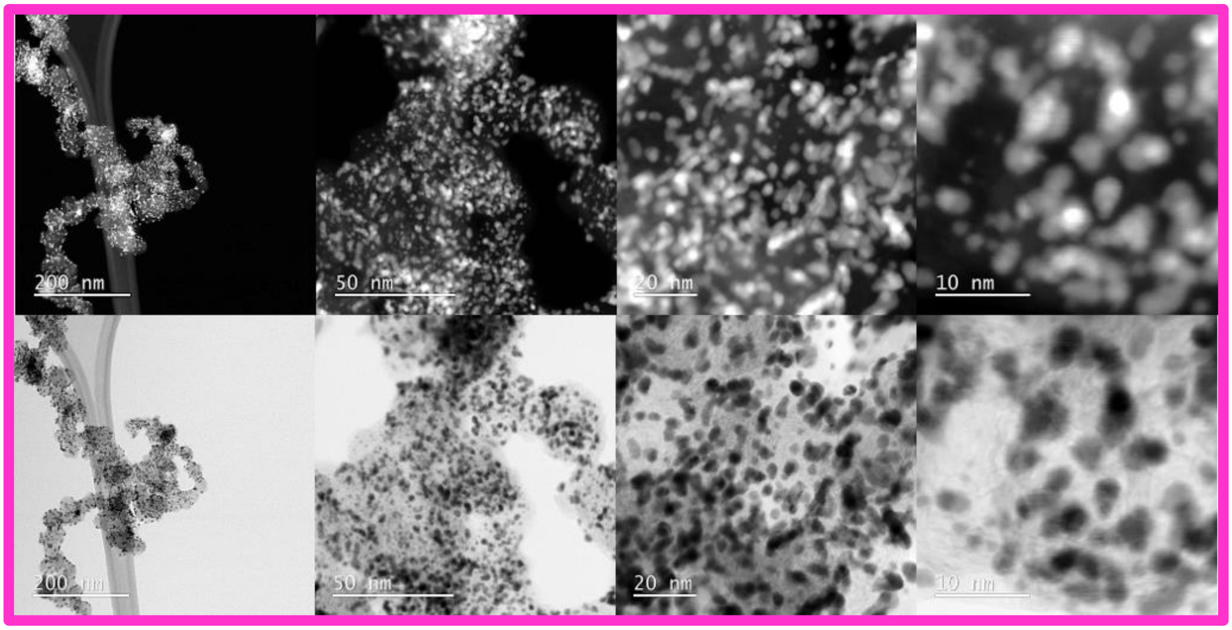

Figure S9. ADF and BF STEM images of Pt+Co/C (C = Ketjen Black EC300J) after Pt NP deposition step *via* GD method.[1]

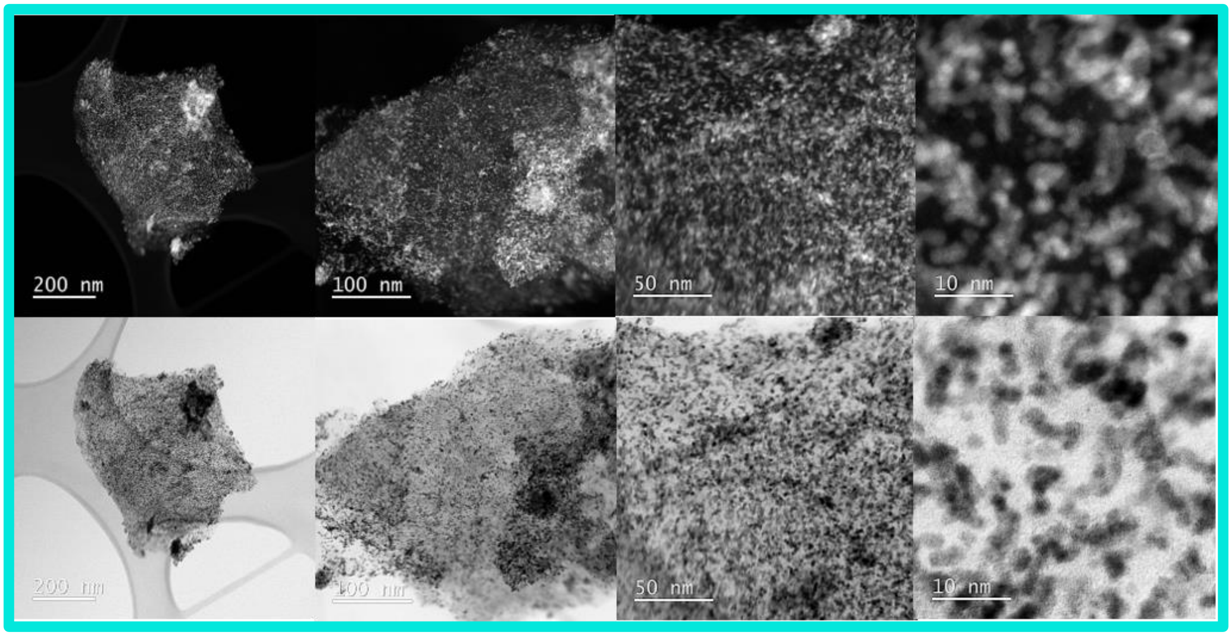

Figure S10. ADF and BF STEM images of PtCu+CuO/rGO after Pt NP deposition step *via* GD method.[1]

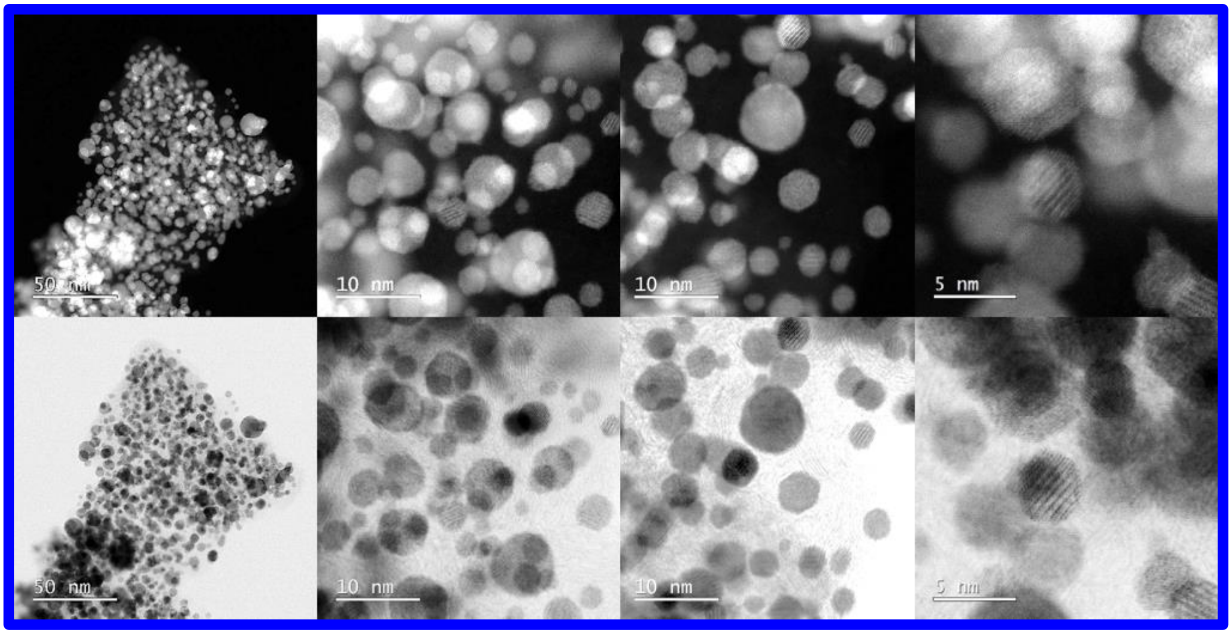

Figure S11. ADF and BF STEM imaging of d-int-Pt-Cu/C electrocatalyst.

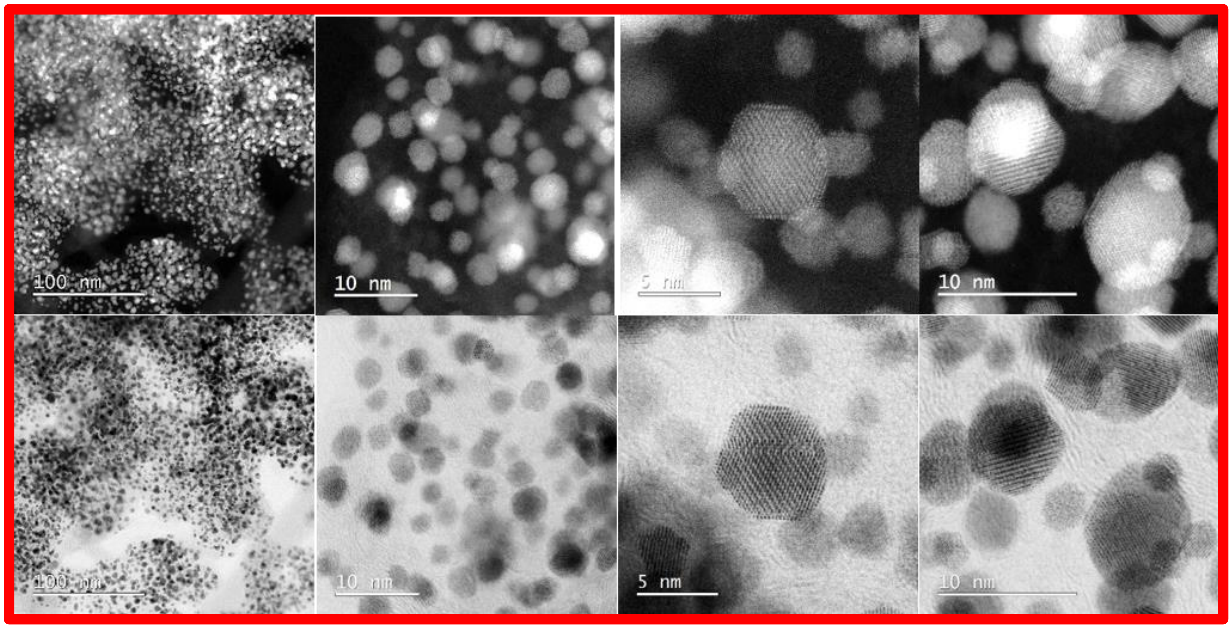

Figure S12. ADF and BF STEM imaging of d-int-Pt-Ni/C electrocatalyst.

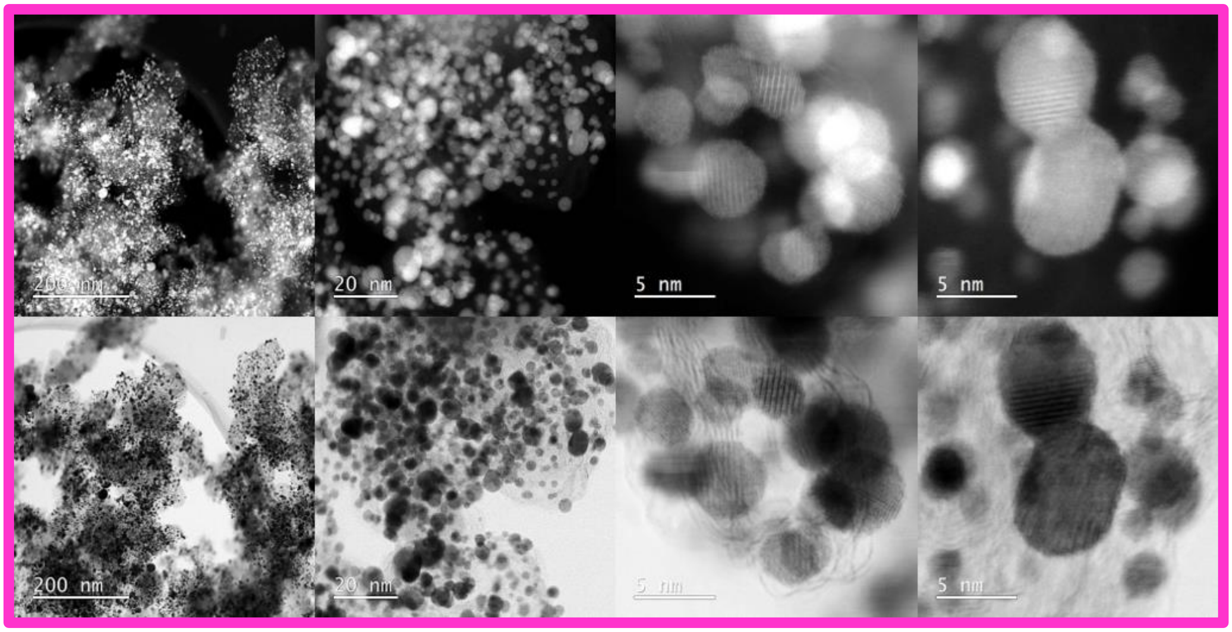

Figure S13. ADF and BF STEM imaging of d-int-Pt-Co/C electrocatalyst.

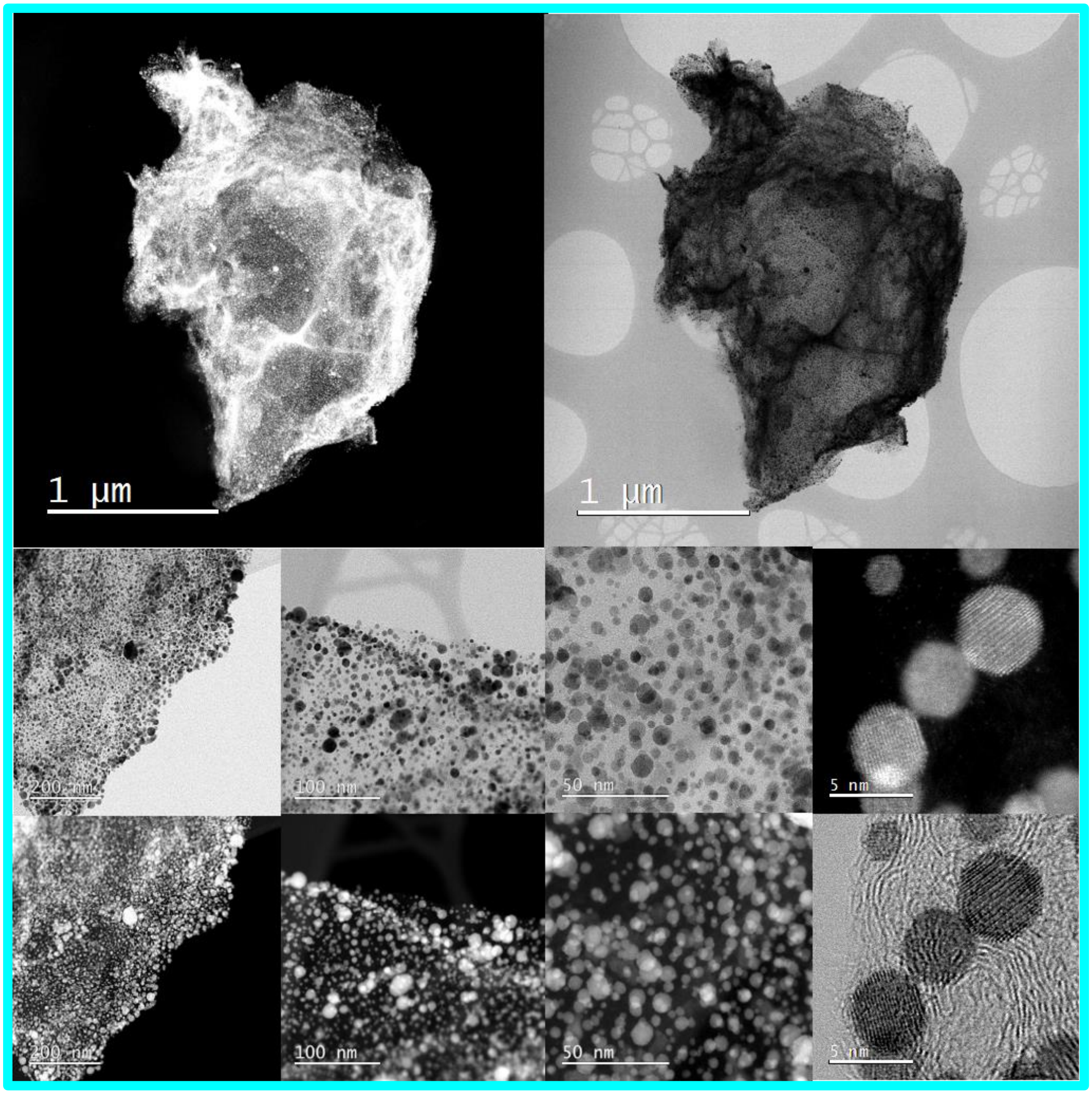

Figure S14. ADF and BF STEM imaging of d-Pt-Cu/C-rGO electrocatalyst.

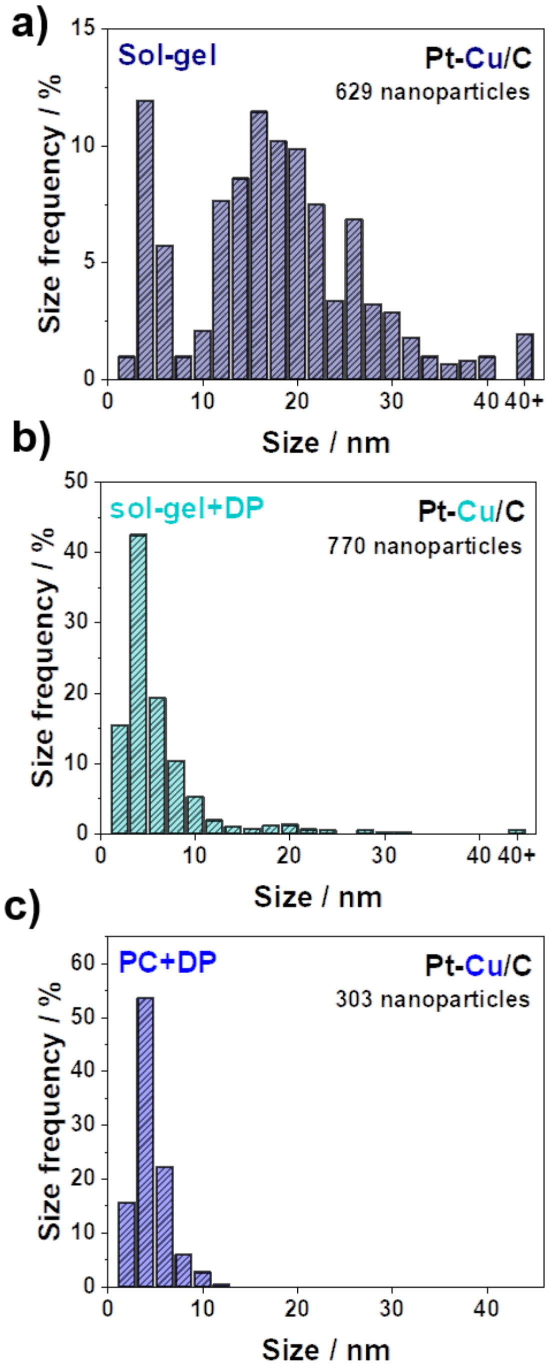

Figure S15. Particle size distribution improvement in the dispersion of Pt-M NPs over carbon upon transitioning from **(a)** sol-gel method for preparation of M/C composites with conventional galvanic displacement for deposition of Pt NPs,[2–5] **(b)** combining sol-gel for preparation of M/C composites with DP method for Pt NP deposition[1,6] and **(c)** utilizing the synergy between PC methodology for the production of M/C composites in combination with DP method for Pt NP deposition (this work).[1,6]

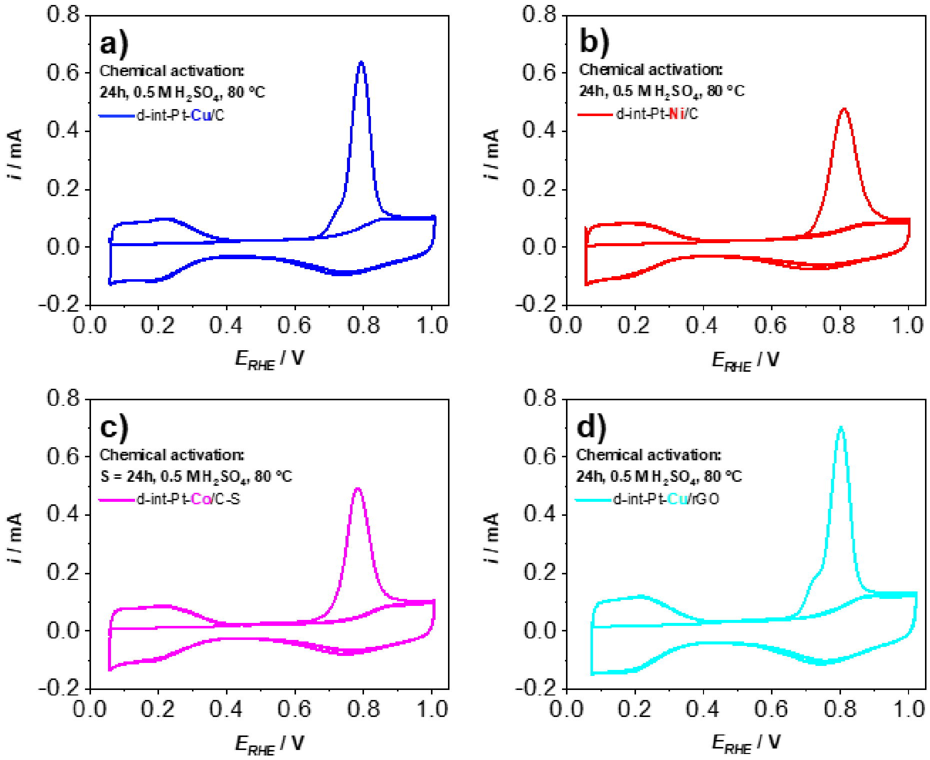

Figure S16. CO-electrooxidation CVs (0.05-1.0 V_{RHE}, 20 mV s^{-1}, no rotation, 0.1 M $HClO_4$, Ar saturated after CO_g adsorption) of **(a)** d-Pt-Cu/C-S, **(b)** d-Pt-Ni/C-S, **(c)** d-Pt-Co/C-S and **(d)** d-Pt-Cu/rGO-S electrocatalysts.

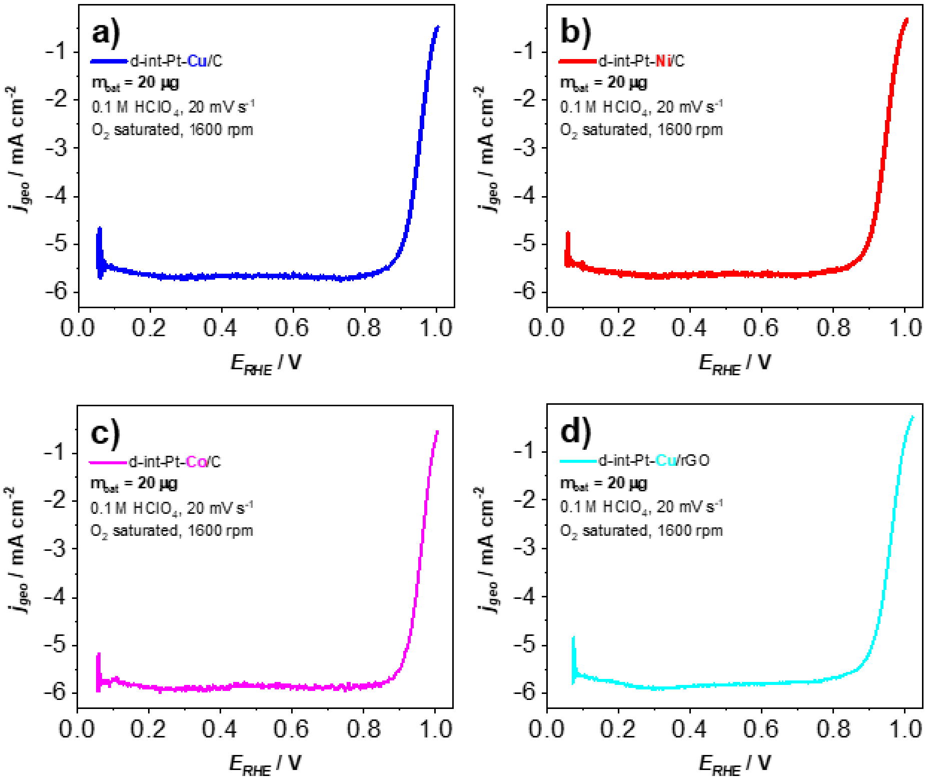

Figure S17. ORR polarization curves (0.05-1.0 V_{RHE}, 20 mV s^{-1}, 1600 rpm, iR and background corrected, 0.1 M $HClO_4$, O_2 saturated) of **(a)** d-Pt-Cu/C-S, **(b)** d-Pt-Ni/C-S, **(c)** d-Pt-Co/C-S and **(d)** d-Pt-Cu/rGO-S electrocatalysts.

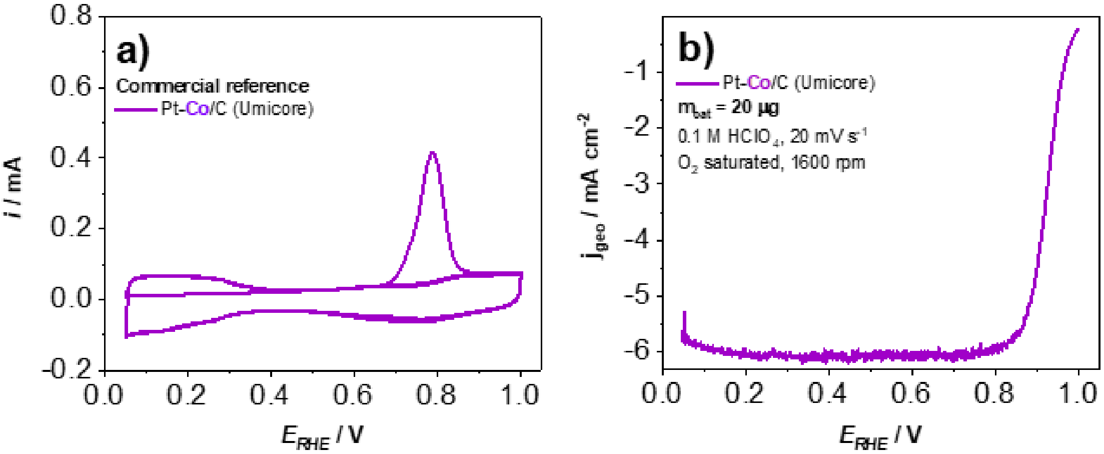

Figure S18. CO-electrooxidation CV (0.05-1.0 V_{RHE}, 20 mV s^{-1}, no rotation, 0.1 M $HClO_4$, Ar saturated after CO_g adsorption) and ORR polarization curve (0.05-1.0 V_{RHE}, 20 mV s^{-1}, 1600 rpm, iR and background corrected, 0.1 M $HClO_4$, O_2 saturated) of Pt-Co/C electrocatalyst from Umicore (Elyst Pt30 0690) with 27 wt% Pt and approximately 3 wt% Co.

REFERENCES

(1) Gatalo, M.; Bele, M.; Ruiz-Zepeda, F.; Šest, E.; Šala, M.; Kamšek, A. R.; Maselj, N.; Galun, T.; Jovanovič, P.; Hodnik, N.; Gaberšček, M. A Double-Passivation Water-Based Galvanic Displacement Method for Reproducible Gram-Scale Production of High-Performance Platinum-Alloy Electrocatalysts. *Angew. Chemie* **2019**, *131* (38), 13400–13404. https://doi.org/10.1002/ange.201903568.

(2) Zhang, J.; Sasaki, K.; Sutter, E.; Adzić, R. R. Stabilization of Platinum Oxygen-Reduction Electrocatalysts Using Gold Clusters. *Science* **2007**, *315* (5809), 220–222.

(3) Zhang, J.; Vukmirović, M. B.; Xu, Y.; Mavrikakis, M.; Adzić, R. R. Controlling the Catalytic Activity of Platinum-Monolayer Electrocatalysts for Oxygen Reduction with Different Substrates. *Angew. Chemie* **2005**, *117* (14), 2170–2173. https://doi.org/10.1002/ange.200462335.

(4) Gong, K.; Su, D.; Adzić, R. R. Platinum-Monolayer Shell on AuNi0.5Fe Nanoparticle Core Electrocatalyst with High Activity and Stability for the Oxygen Reduction Reaction. *J. Am. Chem. Soc.* **2010**, *132* (41), 14364–14366. https://doi.org/10.1021/ja1063873.

(5) Sasaki, K.; Naohara, H.; Choi, Y.; Cai, Y.; Chen, W.-F.; Liu, P.; Adzić, R. R. Highly Stable Pt Monolayer on PdAu Nanoparticle Electrocatalysts for the Oxygen Reduction Reaction. *Nat. Commun.* **2012**, *3*, 1115. https://doi.org/10.1038/ncomms2124.

(6) Gatalo, M.; Hodnik, N.; Gaberšček, M.; Bele, M. Method for Preparation of a Supported Noble Metal-Metal Alloy Composite, and the Obtained Supported Noble Metal-Metal Alloy Composite. PCT/EP2020/057334, 2020.
